# Combined femoral and tibial component total knee arthroplasty device rotation measurement is reliable and predicts clinical outcome

**DOI:** 10.1186/s10195-023-00718-2

**Published:** 2023-08-03

**Authors:** José A. Hernández-Hermoso, Lexa Nescolarde, Federico Yañez-Siller, Juan Calle-García, Damian Garcia-Perdomo, Ricard Pérez-Andres

**Affiliations:** 1 Department of Orthopedic Surgery and Traumatology, Hospital Universitari Germans Trias i Pujol, Carretera Canyet s/n, 08916 Badalona, Barcelona Spain; 2 Department of Surgery, Faculty of medicine, Universitat Autònoma de Barcelona, Campus UAB, 08913 Bellaterra, Spain; 3 Biomedical Engineering, Department of Electronic Universitat Politècnica de Catalunya, Barcelona, Spain; 4 Department of Radiology, Hospital, Universitari Germans Trias i Pujol, Carretera Canyet s/n, 08916 Badalona, Barcelona Spain; 5 Department of Medicine, Faculty of Medicine, Universitat Autònoma de Barcelona, Campus UAB, 08913 Bellaterra, Spain

**Keywords:** Total knee arthroplasty, Tomography, X-ray computed, Rotational alignment, Observer variation, Reproducibility of results, ROC curve

## Abstract

**Background:**

The optimal total knee arthroplasty (TKA) rotational alignment and how best to obtain and measure it are debatable. The aim was to analyse the reliability of the Berger femoral, three different tibial and four different combined two-dimensional computer tomography (2D-CT) TKA component rotation measurements, and to ascertain which rotational values best predict a successful clinical outcome.

**Methods:**

The 2D-CT scans were obtained post-operatively on 60 patients who had TKA. We determined one femoral [Berger’s femoral angle (BFA)], three tibial [Berger’s tibial angle (BTA), anatomical tibial angle (ATA) and bimalleolar posterior tibial component angle (BM_PTCA)] and four combined [transepicondylar posterior tibial component angle (TE_PTCA), bicondylar posterior tibial component angle (BC_PTCA, transepicondylar bimalleolar angle (TE_BM) and bicondylar bimalleolar angle (BC_BM)] TKA rotation angles. We made all measures in 23 patients twice by three observers and determined inter- and intra-observer agreement using the Bland–Altman plot method. We analysed measures of 55 patients using the area under the ROC curve (AUC) analysis to ascertain the discriminative capacity of BFA, ATA, TE_PTCA and BC_PTCA for predicting a successful clinical outcome according to the Knee Society Score (KSS) threshold.

**Results:**

ATA showed the smaller inter- and intra-observer average of differences (−0.1° and 1.6°, respectively) of the studied methods followed by BFA (−0.9° and 1.4°), TE_PTCA (−2.1° and 2.7°) and BC_PTCA (−0.5° and 1.8°). BFA (−4° to 2.1° and −6.1° to 8.8°) and BC_PTCA (−4.4° to 3.4° and −7.9° to 4.4°) showed the narrower inter- and intra-observer limits of agreement. A TKA device rotation (BC_PTCA) < 0.8° of external rotation (ER) predicted a KSS and KSS knee successful outcome, and < 3.8° ER for KSS functional (AUC = 0.889; 0.907 and 0.764, respectively). BFA and ATA < 0.9° ER and < 3.9° internal rotation (IR) predicted a successful KSS knee outcome (AUC = 0.796 and 0.889, respectively).

**Conclusion:**

The ATA tibial component rotation measurement was the most reliable of those studied. BFA, TE_PTCA and BC_PTCA were reliable measures for TKA femoral and combined rotation. The presence of a minimal rotation between the TKA components (BC_PTCA) and a small femoral ER or tibial IR predicted a successful KSS outcome.

*Level of evidence* II.

## Introduction

Rotational alignment has a direct impact on patellar stability [1, 2], knee flexion stability [3, 4], range of motion [3] and polyethylene wear [5]. Thus, in the absence of infection, instability and coronal or sagittal malalignment, malrotation may be a cause of poor outcome and may be a possible indication for total knee arthroplasty (TKA) revision [2, 6–9].

The clinical validity of rotational component TKA measurement methods are dependent on their reproducibility and their correlation with clinical outcome. The optimal TKA rotational alignment for a given patient [10] and how best to obtain and measure it [11] are debatable. Femoral component rotational alignment, but not tibial, can be analysed by plain radiographs with comparable accuracy to computer tomography (CT) [12]. CT scans are the method of choice to assess tibial TKA component rotation [9, 13]. However, measurement of TKA component rotation using different anatomical two-dimensional (2D) and three-dimensional (3D) CT landmarks is challenging, especially for the tibial component [9, 11, 14, 15].

Many authors [3, 6, 7, 13, 16–18] use the Berger method [1], referenced from the surgical transepicondylar axis to establish femoral component rotation. In contrast, there is no consensus about the best method to measure the tibial rotation [6, 11, 14, 19] or combine rotation. Inter- and intra-observer reliability of CT scans, especially for tibial rotation, give conflicting results [7, 8, 14, 18] mainly due to inter-individual variability in anatomical landmarks and difficulty in identification [14, 20] and/or a poorly defined technique for leg positioning during scanning that may influence the location of the landmarks [21].

The degree of TKA component malrotation that can cause clinical problems is debatable [6, 10]. The incidence and extent of internal tibial component rotation are reportedly greater than those for femoral rotation [3, 22]. It appears that combined [1, 10] or isolated femoral [23, 24] and tibial [3, 22] internal component rotation is clinically less well tolerated than external rotation and may be related to pain [1, 22–24] and an increased failure rate [25]. However, it is not known whether isolated femoral or tibial component rotation is more or less related to clinical outcome than combined rotation, due to a possible additive or compensatory effect of the combined rotation.

The rotation between femoral and tibial TKA components, that we define as device rotation, may differ from the arithmetic result (addition or subtraction) of the combination of isolated femoral and tibial component rotations determined with respect to anatomical landmarks. The difference may be due to inter-individual anatomic rotational variability and the possible deformation of the viscoelastic knee soft tissue envelope that may permit maintain the device rotation with different isolated anatomical femoral and tibial rotations.

We had two purposes: (1) to analyse the reproducibility of the Berger femoral, three different tibial and four different combined femoral and tibial 2D-CT TKA component rotation measurement methods and (2) to ascertain the rotation values using the most reproducible methods that discriminate between a good or poor clinical outcome based on the Knee Society Score (KSS). We hypothesize 2D-CT TKA rotational measurements based on well-defined device landmarks will be more reliable and the combined TKA rotation will better discriminate between KSS good or poor clinical outcomes than the isolated femoral and tibial component rotation.

## Patients and methods

### Study design

We performed a prospective, non-randomized, study in 60 patients with painful primary osteoarthritis, who were non-responsive to non-operative treatment and underwent TKA. We excluded patients who had previous fractures of the femur or tibia, or who had previous tibial or femoral osteotomy to eliminate any degree of malrotation. Patients who met the requirements and agreed to participate were consecutively recruited from 2015 to 2017. The procedures followed were in accordance with the ethical standards of the Helsinki Declaration of 1975, as revised in 2000. The local ethics committee approved the study (approval number AC-14-033) and all participants provided written informed consent. Five patients were ultimately excluded from the final clinical evaluation due to acute post-operative infection in two, and severe worsening of Parkinson disease, foot ischaemia, and stroke in three others. The study was conducted in two parts.

### Part 1: Inter- and intra- observer reliability

Twenty-three patients were selected by simple random sampling [26] from opaque sealed envelopes shuffled from the total group. The average age was 71 ± 8 years, average body mass index (BMI) was 30.8 ± 4.2 kg/m^2^, 15 were female and 12 had right TKA. The American Society of Anesthesiology score (ASA) [27] was type II, III and IV in 60.9%, 30.4% and 8.7%, respectively. Pre-operative HKA mechanical axis was measured on weight-bearing hip–knee–ankle lower limb radiographs using a semiautomatic software system (RAIM viewer^®^ version 2.5.0.511). The HKA mechanical lower limb axis average was 5.9° ± 9.0° (2.0°–9.8°).

Three blinded observers [two orthopaedic surgeons (FYS, JCG) and one musculoskeletal radiologist (DGP)] not involved in the surgical procedures reviewed and analysed TKA rotational component angles in the CT images. All reviewers had a 4 h training period before starting the measurements. All measurements were performed twice, with a minimal interval of 6 weeks between each measurement and with no knowledge of previous measurements and those of each other at the time of observation.

### Part 2: Discriminative capacity of TKA component rotation for predicting clinical success

We analysed the full cohort of 55 patients to ascertain the discriminative capacity of TKA component rotation values for predicting a successful clinical outcome. The average age was 71 ± 7 years, average was BMI 30.8 ± 4.2 kg/m^2^, with 36 females and 29 left knees. The ASA score was type I, II, III and IV in 5.2%, 54.5%, 36.4% and 3.6% of the patients, respectively. HKA mechanical lower limb axis average was 5.2° ± 7.7° (3.1°–7.3°).

One blinded observer [orthopaedic surgeon (JCG)] not involved in the surgical procedures or in the clinical follow up reviewed the CT images. That reviewer analysed in all patients the more reliable femoral, tibial and combined TKA rotational component angles, as established in part one of the study.

### Surgical procedure

All patients received a cemented posterior stabilized (PS) Nex-Gen^®^ TKA (Zimmer-Biomet, Warsaw, IN, USA). Antibiotic and thromboembolic prophylaxis was used in all patients. All were operated under tourniquet with a medial parapatellar approach, using a measured resection technique [28] with all instruments and guides provided by Zimmer-Biomet. The proximal tibia cut was made perpendicular to the mechanical axis in the antero-posterior and sagittal planes with the aid of an extramedullary guide. The distal femoral cut, perpendicular to the mechanical axis in the AP and sagittal planes, was performed with the aid of an intramedullary guide. We inserted all femoral component with three degrees of external rotation using a posterior referencing cutting guide. Staged ligamentous releases to balance flexion–extension gaps were performed [29, 30]. Tibial rotation was established in relation to the medial third of the anterior tibial tuberosity. Patellar resurfacing was performed in all procedures by subluxing the patella. All patients underwent the same post-operative protocol.

### Clinical assessment

One blinded observer (FYS) not involved in the surgical procedures examined all patients using the Knee Society Score (KSS) [31] clinical protocol at 1 year post-operative follow up. The KSS_POST is the sum of a clinically rated portion (knee score, KSS_KNEE-POST) that covers pain, range of movement, alignment and stability and a patient reported portion (function score, KSS_FUNCTION_POST) that covers patient’s mobility (walking distance and stairs) and potential walking aids. The KSS score range is from 0 to 100 points for each portion, with higher scores indicating better outcome.

### 2D-CT radiological measurements

Non-contrast material-enhanced helical 2D-CT scans were obtained at 6 months post-operatively using a LightSpeed VCT 64 scanner (General Electric, Hino, Japan). The protocol for CT scan uses 140 kV, 400 mA and 0.6 mm thick axial images obtained at four locations of the knee (the femoral epicondylar axis, the tibial component tray, the proximal tibial plateau and the tibial tubercle) and at the ankle joint. Only the axial imaging of the knee and ankle were considered for this study. Patients were scanned in the supine position with knees in maximum extension and legs fixed in neutral rotation as determined by facing the patella forward. regardless of the foot position. The knee was scanned from the superior patellar margin to the bottom of the anterior tibial tuberosity and 2–3 cm on either side of the tibiotalar joint. For the two regions the *x*, *y* and *z* axes were kept fixed for the duration of the scan to maintain the relative position of the two regions with respect to each other.

We used image processing software dedicated to DICOM images, with a measuring tool within the Alma Workstation 4.2.3.0 (Alma Medical Imaging, Barcelona, Spain) program to measure distances and angles. External rotation (ER) of the component was considered to be a positive value and internal rotation (IR), a negative value.

**Fig. 1:**
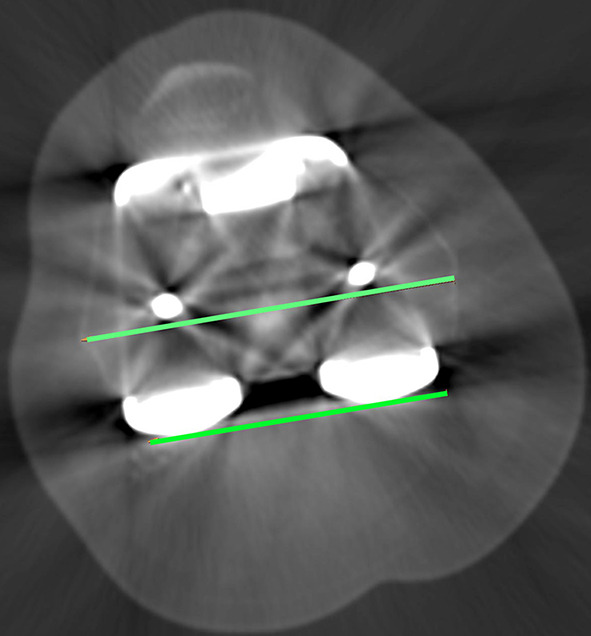
The Berger’s femoral component rotational angle (BFA). Axial CT image showing measurement of the rotation of the femoral component using the surgical transepicondylar axis and the posterior femoral condylar axis. The surgical transepicondylar axis is a line drawn between the lateral epicondylar prominence and the medial sulcus of the medial epicondyle in a slice where the two landmarks were visible. The posterior femoral condylar axis is a line drawn tangential to the posterior surface of the two condyles of the TKA femoral component

We determined Berger’s femoral component rotation angle (BFA) using the method described by Berger [1] (Fig. 1). To simplify the measurement, we, as other authors [3, 13] do not apply differing male and female corrections of native rotation from the posterior condylar angle (e.g. 0.3° ± 1.2° IR in females and 3.5° ± 1.2° IR in males).

**Fig. 2:**
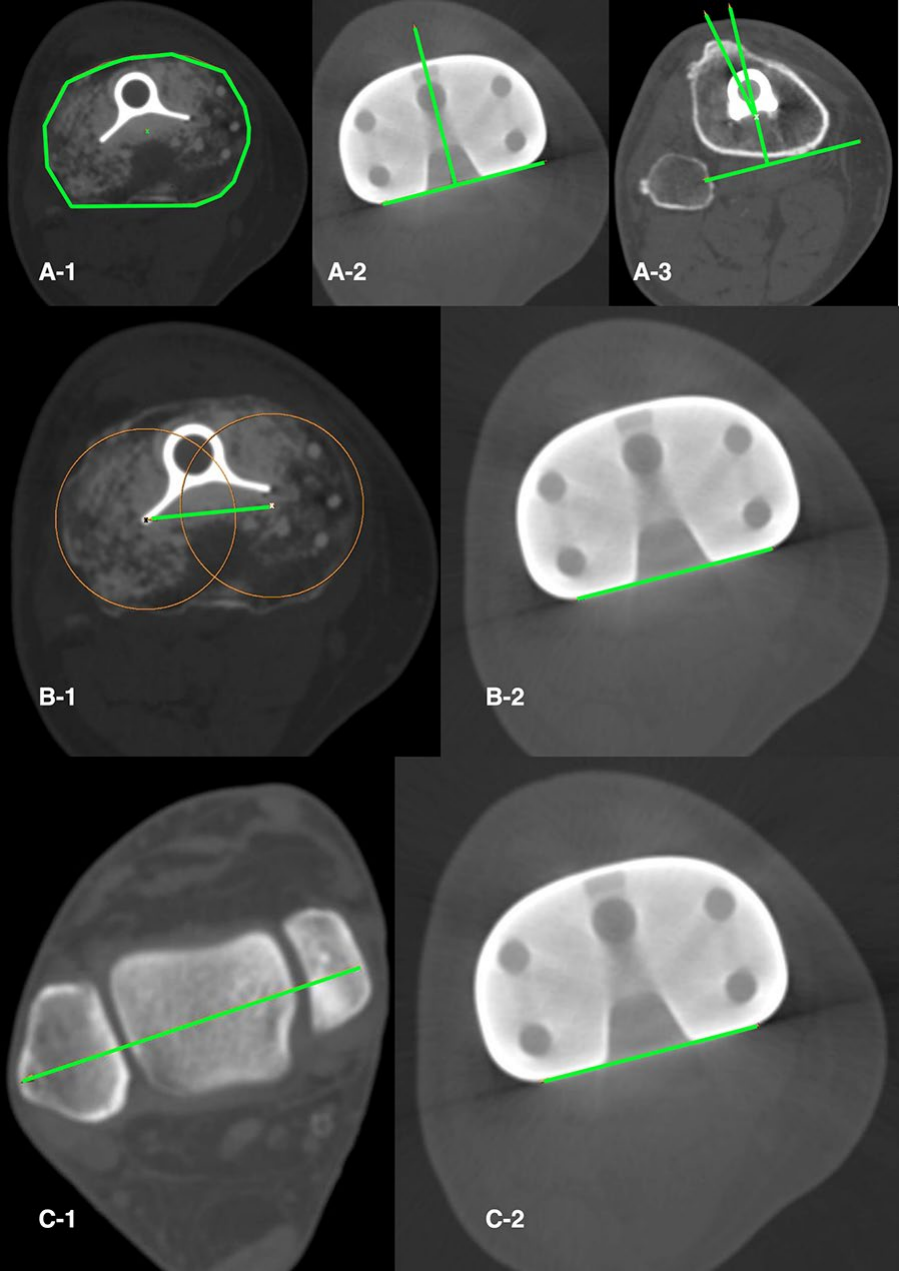
TKA tibia component rotation measures. **A** Beger’s tibial angle (BTA) formed between the line that connects the geometric centre of the tibial plateau and the tip of the tibial tubercle and the anteroposterior tibial component axis. The geometric centre of the tibial plateau measured in the first CT slice just under the metal tray (A-1) was located and axially transposed to CT slice at the level of the tibial tubercle (A-3). Then, the geometric centre of the tibial plateau and the tip of the tibial tubercle are connected (A-3). The anteroposterior tibial component axis is drowned in a single axial scan through the tibial component perpendicular to the posterior tibial component axis (A-2) and transposed to the CT slice at the level of the tibial tubercle. The posterior tibial component axis was a line tangential to the posterior TKA tibial plateau. The tip of the tibial tubercle is 18° (± 2.6°) externally rotated from the native tibial articular surface, the tibial component was considered neutral (0°) when internally rotated 18° in relation to the tip of the tibial tuberosity. **B** Anatomic tibial angle (ATA) formed between the anatomic tibial axis (B-1) and the posterior tibial component axis (B-2). The anatomic tibial axis is the line defined by the geometric centre of the lateral tibial plateau and the geometric centre of medial tibial plateau measured in the first tibial CT slice just under the TKA tibial component (B-1). **C** Bimalleolar posterior tibial component angle (BM_PTCA) is the angle formed by the transmalleolar axis (C-1) between the centre of the medial malleolus and the centre of the fibula in an axial slice situated at the ankle centre and bisecting the malleoli and the posterior tibial component axis (C-2)

We assessed three tibial component rotation measurement techniques. First, Berger´s tibial angle (BTA) (Fig. 2A), using the method described by Berger [1]. Second, the anatomic tibial angle (ATA) (Fig. 2B), using the method described by Cobb [14]. Third, the bimalleolar posterior tibial component angle (BM_PTCA) (Fig. 2C), formed by the transmalleolar axis and the posterior tibial component axis. The transmalleolar axis is a line between the centre of the medial malleolus and the centre of the fibula in an axial slice situated on the ankle centre and bisecting the malleoli [32] (Fig. 2C-1). The posterior tibial component axis was a line tangential to the posterior TKA tibial plateau.

**Fig. 3:**
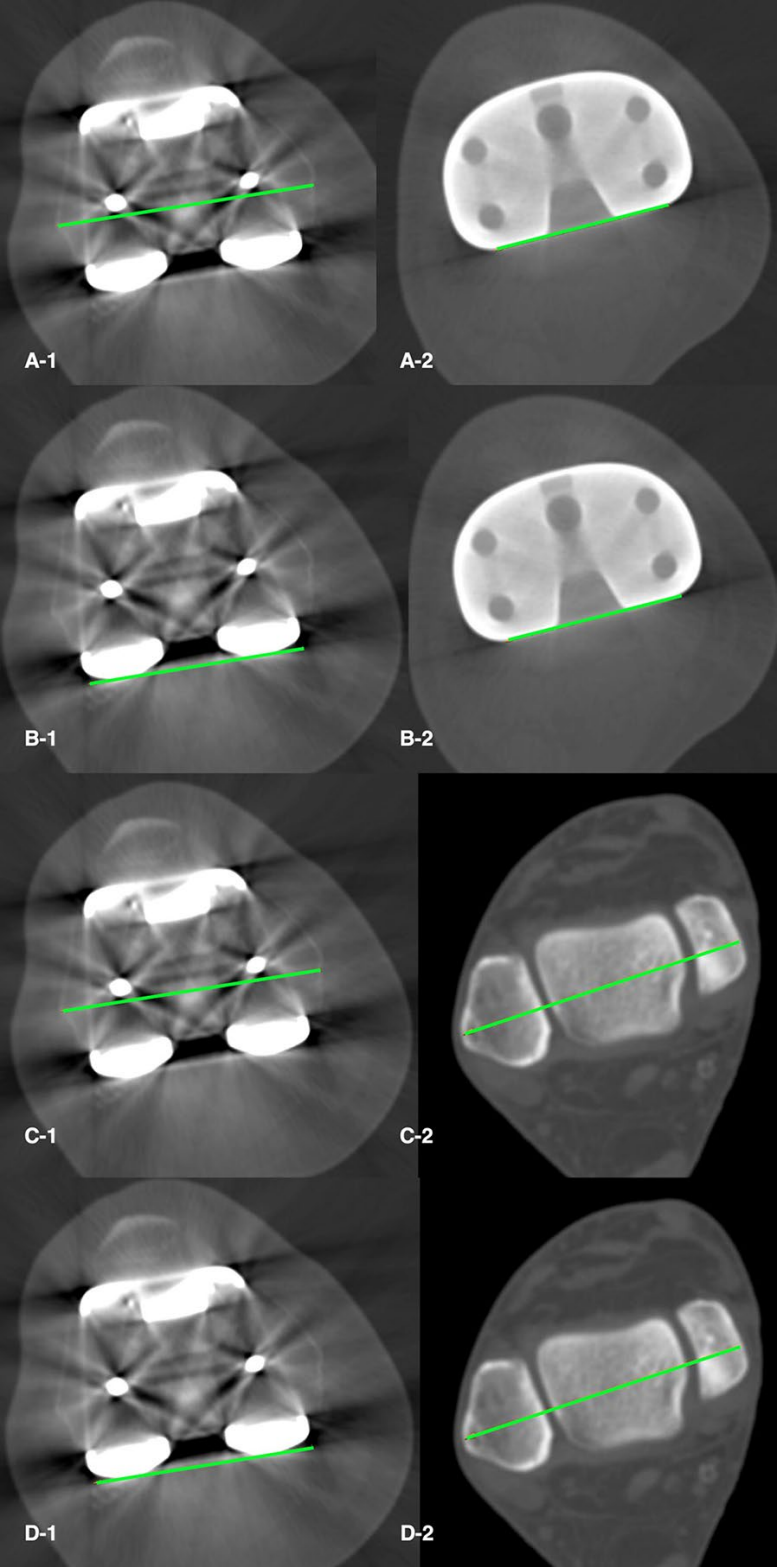
TKA combined rotation measures. **A** Transepicondylar posterior tibial component angle (TE_PTCA) is the angle between the surgical transepicondylar axis (A-1) and the posterior tibial component axis (A-2). **B** The bicondylar posterior tibial component angle (BC_PTCA) is the angle formed by the posterior femoral condylar axis (B-1) with the posterior tibial component axis (B-2). **C** The transepicondylar bimalleolar angle (TE_BM) is the angle between the surgical transepicondylar axis (C-1) and the transmalleolar axis (C-2). **D** The bicondylar bimalleolar angle (BC_BM) is the angle between the posterior femoral condylar axis (D-1) and the transmalleolar axis (D-2)

We evaluated four combined femoral and tibial TKA rotation measurement methods. First, the transepicondylar posterior tibial component angle (TE_PTCA) (Fig. 3A), formed by the surgical transepicondylar axis and the posterior tibial component axis. The surgical transepicondylar axis was a line drawn between the lateral epicondylar prominence and the medial sulcus of the medial epicondyle in a slice where the two landmarks were visible. Second, the bicondylar posterior tibial component angle (BC_PTCA) (Fig. 3B), formed by the posterior femoral component axis and the posterior tibial component axis. The posterior femoral component axis was a line drawn tangential to the posterior surface of the two condyles of the TKA femoral component. This angle measures the rotation between the TKA femoral and tibial components independently from anatomic landmarks, it is the device rotation. Third, the transepicondylar bimalleolar angle (TE_BM) (Fig. 3C), formed by the surgical transepicondylar axis and the transmalleolar axis. Fourth, the bicondylar bimalleolar angle (BC_BM) (Fig. 3D), formed by the posterior femoral component axis and the transmalleolar axis.

### Statistical analyses

The normal distribution of all the data was confirmed using the Kolmogorov–Smirnov test and the homogeneity of variances by Levene’s test.

A sample size calculation could not be done at the beginning of the study because we did not have data of the new angles studied, and it was performed at the end to determine whether the study had adequate power. The sample size calculation utilized previously published differences between methods for the already described femoral and tibial rotational angles [8] and the mean ± SD of the obtained values in the present study for the new tibial and combined rotational angles that lack of reference data. A desired sample size between 12 and 23 patients was calculated with an α level of 0.05 and a β level of 0.20 (80% power), using MedCalc statistical software version 19.0.3 (MedCalc Software Bvba, Ostend, Belgium) to assess agreement between the observers using the Bland–Altman plot analysis. The expected mean of differences, the expected SD of differences and the maximum allowed difference between methods was set at −0.9°, 1.6° and 6° for BFA; −4.1°, 3.0° and 12.0° for BTA; 0.1°, 4.0° and 16.0° for ATA; 1.1°, 4.3° and 17.0° for BM_PTCA; −0.5°, 2.8° and 11.0° for TE_PTCA; − 0.5°, 2.0° and 7.0° for BC_PTCA; − 2.0°, 2.8° and 11.0° for TE_BM and −2.8°, 3.0° and 12.0° for BC_BM, respectively.

The inter-and intra-observer agreement between two measurements was assessed using the Bland–Altman plot method [33]. For inter-observer agreement, Observer 3 (JCG) was taken as reference and the first rotational measure was used. For intra-observer agreement the first and second rotational measure of the three observers was taken. The limits of agreement (LOAs) were set, within which 95% of the differences between one measurement and the other are included were calculated.

The area under the ROC curve (AUC) analysis [34] was used to establish the discriminative capacity of BFA, ATA, TE_PTCA and BC_PTCA for predicting a successful clinical outcome according to the established KSS thresholds of 160, 70 and 86 points for KSS_POST, KSS_FUNCTION_POST and KSS_KNEE_POST, respectively [35], for discriminating between patients with or without treatment success following TKA. A test is considered good, very good or excellent when the AUC is 0.75–0.9, 0.9–0.97 or 0.97–1, respectively [34].

The analyses were performed using statistical software IBM^®^ SPSS^®^ version 28.0 (IBM Corp.; Armonk, NY, USA). The statistical significance was set at *p* < 0.05.

## Results

### Inter-observer reliability

**Fig. 4:**
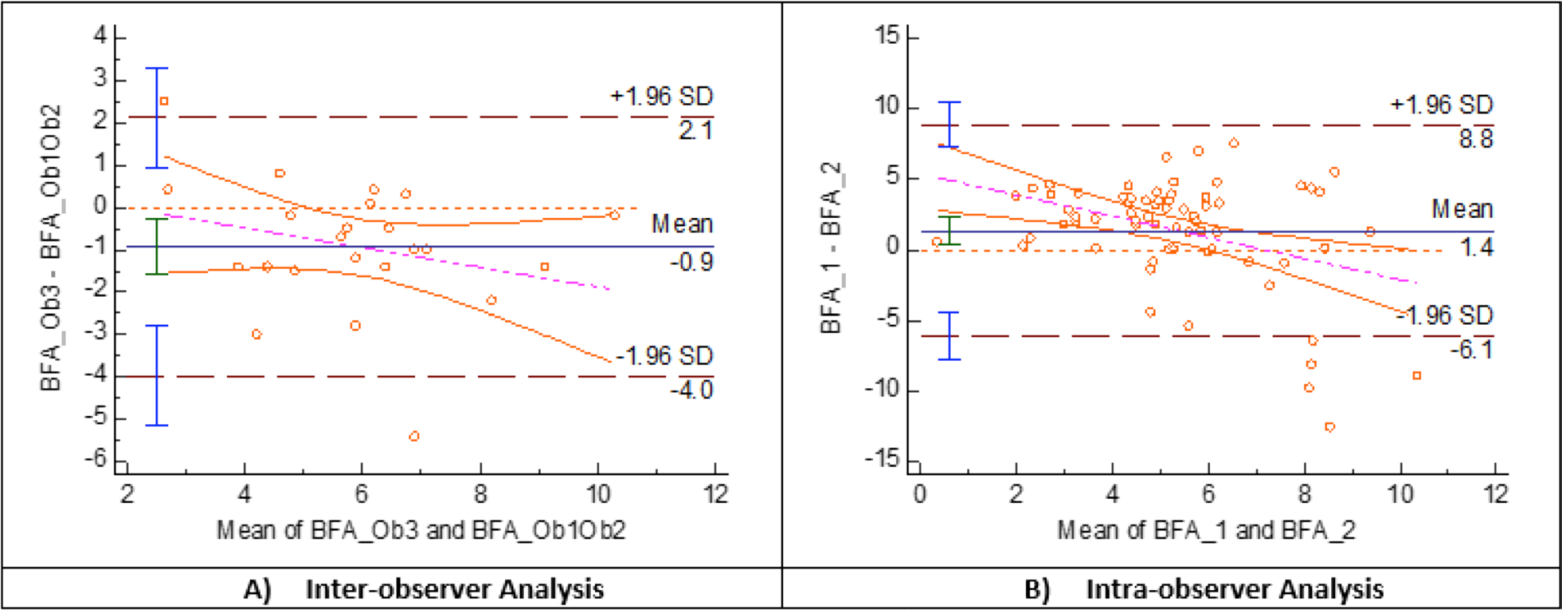
Berger’s femoral component rotational angle (BFA) Band–Altman plot agreement. The inter- **A** and intra-observer **B** agreement between two measurements. For inter-observer agreement, Observer 3 was taken as reference and the first reliable rotational measure was used. For intra-observer agreement, the first and second reliable rotational measure of the three observers was taken

**Fig. 5:**
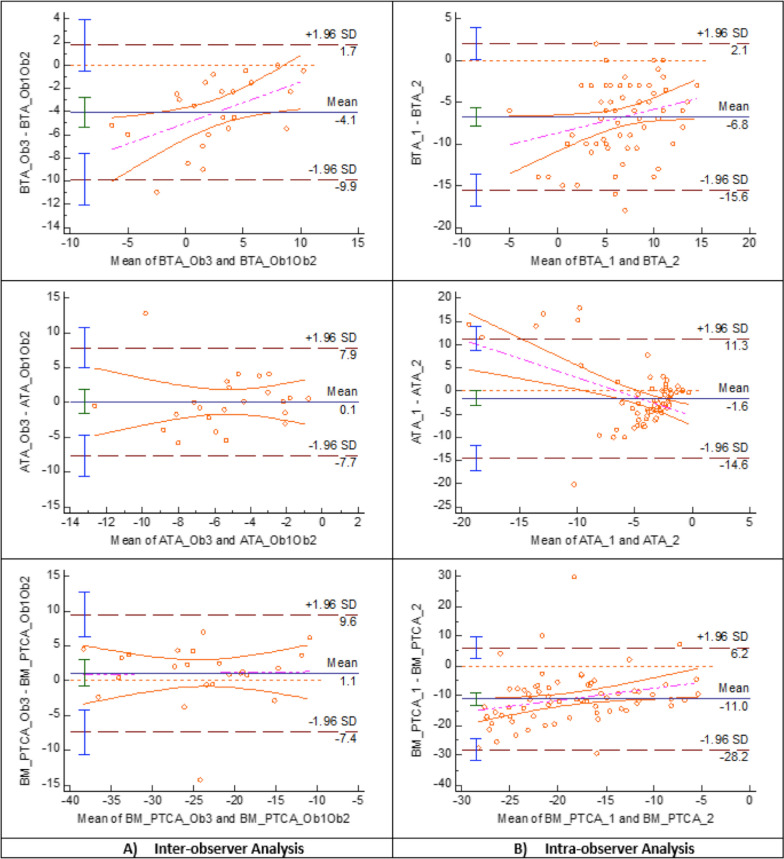
TKA tibia component rotation angles Bland–Altman plot agreement. The inter- **A** and intra-observer **B** agreement between two measurements. *BTA* Berger’s tibial angle, *ATA* anatomic tibial angle, *BM_PTCA* bimalleolar posterior tibial component angle, *Ob1* Observer 1, *Ob2* Observer 2. *Ob3* Observer 3. First measure: 1; second measure: 2

**Fig. 6:**
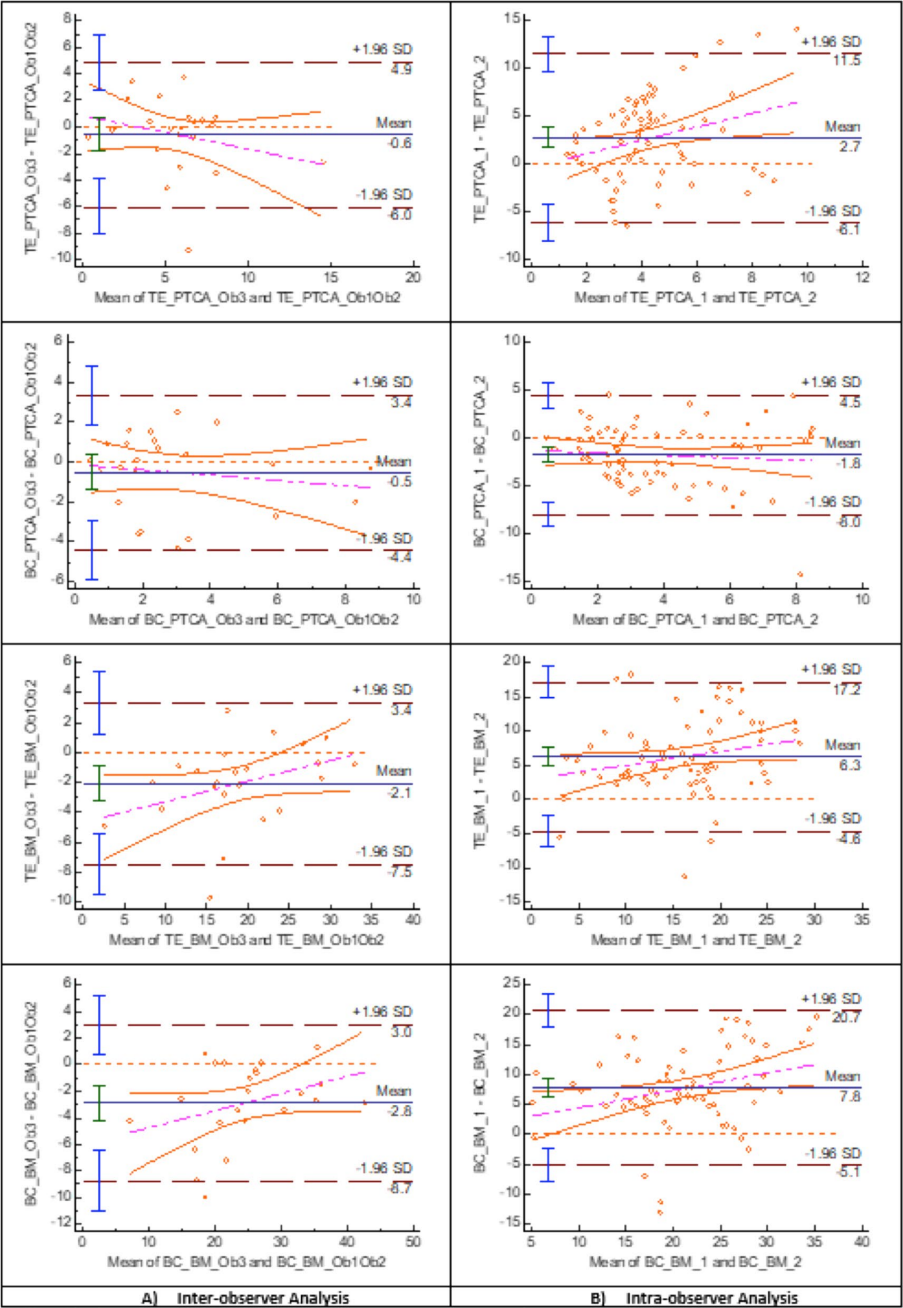
TKA combined rotation angles Bland–Altman plot agreement. The inter- **A** and intra-observer **B** agreement between two measurements. *TE_PTCA* transepicondylar posterior tibial component angle, *BC_PTCA*, bicondylar posterior tibial component angle, *TE_BM* transepicondylar bimalleolar angle, *BC_BM* bicondylar bimalleolar angle, *Ob1* Observer 1, *Ob2* Observer 2, *Ob3* Observer 3 First measure: 1; second measure: 2

Bland–Altman plots showed larger systematic bias between the mean difference of the first measure of each observer in BTA (4°), TE_BM (−2.1°) and BC_BM (−2.8) than in BFA (−0.9°), ATA (−0.1°), BM_PTCA (−1.1°), TE_PTCA (−0.6°) and BC_PTCA (−0.5°), in which the average of the differences between observers was close to 0°. The limits of agreement were narrower for BFA (−4° to 2.1°), TE_PTCA (−6° to 4.9°) and BC_PTCA (−4.4° to 3.4°) than for ATA (−7.9° to 7.7°) and BM_PTCA (−9.6° to 7.4°). The line of equality was within the 95% confidence interval (CI) of the mean difference between observers in ATA, BM_PTCA, TE_PTCA and BC_PTCA and very close to being within in BFA (Figs. 4, 5 and 6) (Table 1).

**Table 1:** Femoral, tibial and knee TKA component rotation angles measurements

	Radiological measurements (grades)	Observer 1		Observer 2		Observer 3	
		First measure	Second measure	First measure	Second measure	First measure	Second measure
Femoral rotation	BFA	6.3 ± 1.7 (5.5–7.1)	3.7 (3.3) (0.2–12.2)	6.7 ± 2.5 (5.6–7.8)	4.6 (3.0) (0.1–14.8)	5.6 ± 1.7 (4.8–6.3)	3.1 (3.6) (0.4–13.0)
Tibial rotation	BTA	3.4 ± 4.4 1.5–5.4	9.2 ± 2.6 8.0–10.3	6.3 ± 3.4 4.8–7.8	14.0 (5.3) 3.0–17.0	0.7 ± 5.2 −1.6 to 3.0	7.9 ± 3.4 6.4–9.4
	ATA	− 5.4 ± 0.6 −6.8 to −4.1	−1.7 (1.8) −24.0 to −0.4	−4.8 (4.8) − 24.0 to −0	−1.3 (8.6) −26.5 to −0	−5.4 ± 3.6 −7.0 to −3.8	−1.8 ± 1.2 −2.3 to −1.3
	BM_PTCA	− 23.1 ± 9.1 −27.2 to −19.1	−13.9 ± 6.1 −16.6 to −11.2	−25.6 ± 8.8 −29.5 to −21.7	−14.6 ± 6.6 −17.3 to −11.4	−23.4 ± 8.1 −27.0 to −19.8	−12.3 ± 6.4 −15.1 to −9.4
Knee rotation	TE_PTCA	6.0 ± 3.2 (4.6–7.4)	3.0 ± 2.3 (2.0–4.0)	6.0 ± 3.9 (4.3–7.8)	2.6 (4.2) (0.1–9.4)	5.3 ± 2.9 (4.0–6.6)	2.2 (3.7) (0.1–9.7)
	BC_PTCA	2.4 (2.4) (0.5–8.8)	4.6 ± 2.5 (3.5–5.6)	2.9 (5.0) (0.2–10.1)	4.8 (3.1) (0.1–15.3)	2.0 (3.6) (0.1–8.6)	4.5 ± 2.6 (3.4–5.6)
	TE_BM	19.0 ± 7.5 (15.7–22.3)	13.1 ± 6.0 (10.5–15.7)	20.5 ± 6.9 (17.5–23.6)	13.6 ± 6.5 (10.8–16.4)	17.8 ± 8.1 (14.2–21.5)	12.2 ± 6.4 (9.5–15.0)
	BC_BM	25.2 ± 8.5 (21.5–29.0)	17.5 ± 6.5 (14.7–20.3)	27.0 ± 7.9 (23.5–30.5)	18.2 ± 7.0 (15.2–21.2)	23.3 ± 9.0 (19.4–27.3)	16.5 ± 6.3 (13.8–19.2)

BFA, Berger’s femoral angle; BTA, Berger’s tibial angle; ATA, anatomical tibial angle; BM_PTCA, bimalleolar posterior tibial component angle; TE_PTCA, transepicondylar posterior tibial component angle; BC_PTCA, bicondylar posterior tibial component angle; TE_BM, transepicondylar bimalleolar angle; BC_BM, bicondylar bimalleolar angle

Description of 2D-CT TKA component rotational radiological measurements of the first and second measure for Observer 1, Observer 2 and Observer_3. The variables normally distributed are shown as mean ± SD, 95% confidence interval for mean (lower bound and upper bound), while non-normally distributed data are shown as statistic median (interquartile range, IQR) and minimum–maximum

### Intra-observer reliability

Bland–Altman plots showed larger systematic bias between the mean difference of the first with the second measure of the observers in BTA (6.8°), BM_PTCA (11.0°), TE_BM (6.3°) and BC_BM (7.8°) rotational alignment measurements than in BFA (1.4°), ATA (1.6°), TE_PTCA (2.7°) and BC_PTCA (1.8°) in which the average of the differences between the first and second measure was close to 0°. The limits of agreement were narrower for BFA (−6.1° to 8.8°) and BC_PTCA (−7.9° to 4.4°), than for ATA (−11.5° to 14.7°) and TE_PTCA (−6° to 11.5°). The line of equality was within the 95% confidence interval (CI) of the mean difference between observers in ATA and was very close to be within in BFA and BC_PTCA (Figs. 4, 5 and 6) (Table 1).

### Discriminative capacity of TKA component rotation for predicting KSS clinical outcome

**Fig. 7:**
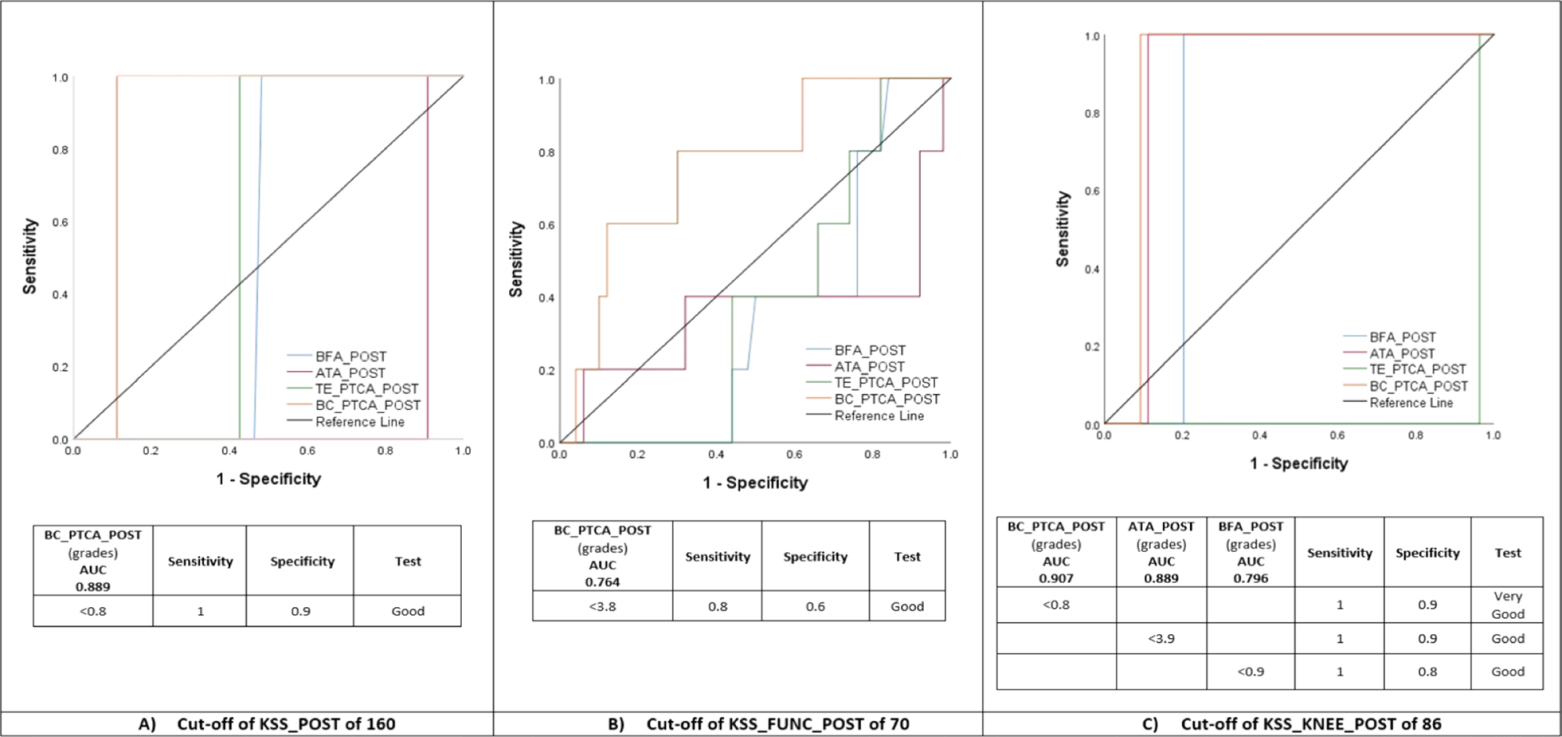
TKA component rotation prediction of successful clinical outcome. AUC was used to establish the discriminative capacity of BFA (Berger’s femoral angle), ATA (anatomical tibia angle), TE_PTCA (transepicondylar posterior tibial component angle) and BC_PTCA (bicondylar posterior tibial component angle) for predicting a successful clinical outcome according to the established KSS (Knee Society Score) thresholds of 160, 70 and 86 points for KSS_POST, KSS_FUNCTION_POST and KSS_KNEE_POST, respectively, for discriminating between patients with or without treatment success following TKA

A small amount of device rotation (BC_PTCA) (< 0.8° ER) was a good cut-off value to predict KSS_POST and KSS_KNEE_POST success (AUC = 0.899 and 0.907, respectively), and < 3.8° ER was a good cut-off value to predict KSS_FUNCTION_POST success (AUC = 0.764). The femur BFA and tibia ATA rotation < 0.9° ER and < 3.9° IR, respectively, were good cut-off values to predict KSS_KNEE_POST success (AUC = 0.796 and 0.889, respectively) (Fig. 7).

## Discussion

In the absence of a reliable and a reproducible method to measure TKA rotation [7, 9, 11, 20, 36, 37] and the absence of a clear definition of what is tolerable rotational TKA alignment [10], it is difficult to establish what malrotation might result in a painful TKA [8, 10] and when to recommend a surgical decision [3].

Our data suggest the ATA method has the best inter- and intra-observer agreement. BM_PTCA, TE_PTCA and BC_PTCA showed a good inter-observer agreement. The BFA method had both inter- and intra-observer agreement close to good, and the BC_PTCA method had an intra-observer agreement close to be good. The most important finding is that a device rotation (BC_PTCA) less than 0.8° ER is good to predict KSS and KSS_KNEE success and less than 3.8° ER is good to predict KSS_FUNCTION success 1 year after a TKA. BFA less than 0.9° ER and ATA less than 3.9° IR showed a good capacity to predict KSS_KNEE success.

### Inter- and intra-observer reliability

Berger´s femoral angle has been widely used [2, 3, 7–9, 13, 16], with a range of reliability ranging from good [8, 13] to poor [7, 9]. We found means values of BFA similar to other studies [7, 9, 13] and an acceptable inter- and intra-observer agreement with narrow agreement limits. Although the identification of the medial sulcus is demanding, as well as the posterior component condyles, due to the metal scatter and superposition with the tibial tray, we believe that the landmarks are well defined and reduce the source of variability.

There is no accepted method for measuring the tibial component rotation [11, 14]. The protocol described by Berger et al. [1] to measure BTA has contrasting studies that report good [7, 8, 11] and poor [19, 38] inter- and intra-observer ICC with no correlation with clinical outcomes [8]. We found poor inter- and intra-observer agreement. The lack of reliability may be due to the transposition of the measurements performed in three different axial CT slices, to the difference in tibial tuberosity anatomies [19, 39] or to a tubercle anatomical position that varied more than any other point in the mediolateral plane [14, 40].

While one study [26] suggests the ATA method has poor inter-observer agreement and a good intra-observer agreement, we found ATA was the more reliable method. In contrast to BTA, the ATA has been less used [41], but may be a less complex measurement, because it requires the transposition of only two axial CT slices, and it is independent from the tibial tubercle positioning and variability [14]. The irregular shape of the tibia cortex under the TKA tibial tray makes it challenging to determine the centre of the medial [14] and the lateral [8] tibial plateau, either of which may cause of variability of the measure. Despite this good agreement, the limits of agreement were slightly greater than previous reported ones [8].

We found good inter-observer agreement but poor intra-observer agreement for the BM_PTCA tibial TKA component rotation with wide limits of agreement. We believe it is especially challenging to select the proper CT slice at the ankle joint to take the measurement [32], and to establish the centre of the tibia-peroneal joint and medial malleolus that may favour variability of the transmalleolar axis.

Few techniques have been described to measure the femoral and tibial TKA component rotation together [6]. The “combined rotation” occurs when femoral and tibial TKA component rotation occur in the same direction [1] and “mismatch rotation” when they occur in the opposite direction [22, 24]. Combined [1, 22, 24] and mismatch [1] TKA rotation have been calculated by arithmetically adding the obtained isolated femoral from tibial rotation. Mismatch TKA rotation has also been calculated by arithmetically subtracting the isolated femoral from tibial rotation [22, 24]. We analysed four methods to establish the combined TKA rotation that avoid combining the variability of two isolated measures. The two methods based on the ankle transmalleolar axis, TE_BM and BC_BM, had poor agreement, perhaps owing to the previously mentioned difficulty of establishing the transmalleolar axis. The TE_PTCA and the BC_PTCA with well-defined TKA device landmarks had good inter-observer agreement and the BC_PTCA intra-observer agreement was close to good.

### Discriminative capacity of TKA component rotation for predicting KSS clinical outcome

The variability of distal femoral rotational anatomy in patients undergoing TKA range from 3.3° to 11° [42]. TKA femoral ER reportedly increases wear and tightness in the popliteus tendon complex while IR may increase stress, wear and subluxation on the patellar implant [10] and tightens the medial flexion gap [3]. With a TKA femoral component ER < 2° the failure rate is 6.75% and with > 5° failure rate is 1.9%, with no TKA required revision within 2°–5° ER [31]. A femoral component IR > 3.9° with respect to the surgical transepicondylar axis may favour unexplained knee pain [24]. We found a TKA femoral component ER less than 0.9°, without corrections by sex, predicted good KSS_KNEE scores.

Tibial TKA component IR > 9° in relation to neutral tuberosity reportedly may relate to pain and limited range of motion (ROM) [3, 10, 22], and even a smaller limit of tolerability of 5.8° of tibial IR has been reported for Nex Gen Legacy posterior stabilized flex fixed bearing TKA [24]. We found a TKA with tibial rotation (ATA) less than 3.9° IR predicted good KSS_KNEE scores.

Combined internal rotation > 8.7° and component rotation mismatch of > 5.6° is a factor in painful TKA [24]. A similar combined or mismatch rotation of the TKA components measured with different anatomic landmarks may have different rotation between TKA components (device rotation) due to the variability of knee rotational anatomy and/or to the laxity or tightness of knee soft tissue envelope. In one cadaver study [43], the TKA component femorotibial matching varied from 2° to 8° ER. Although we used a different reference line in the tibia component our femorotibial matching variation was from 4.5° ER to 8° IR. We realize that a small rotation between TKA components – device rotation – less than 0.8° or 3.8° ER, is important to obtain a good TKA KSS total, function and knee clinical score. We assume that if the device rotation increases it may alter the static and dynamic balance of the knee soft tissue envelope [44] in the transverse plane and may favour a painful TKA, depending on whether the patient adapts to it over time.

The wide limits of agreement of TKA component rotation angles we found is generally consistent with the described variability of component rotation after TKA based on CT findings that range from 25° for the tibial component to 9° for the femoral one and to 12° for the combined rotation [7, 8, 14] This variability may result from a number of measurements found outside the margins considered as recommended for TKA component rotational alignment in an overview of the literature by Gromov et al. [10]. We agree that caution must be applied with measurements on 2D-CT scans when attributing symptoms or indicating a revision surgery for component malalignment [7, 8] due to individual and technical measurement variability.

We note several limitations. First, the short follow-up time (1 year) does not allow us to determine whether these results will change over time, although usually the TKA clinical result do not improve after this period of time. Second, all the measurements were done with a single PS fixed-bearing TKA having a symmetric tibial tray implanted trough a measure resection technique; therefore, our results do not extend to patients with other types of TKA constraint, mobile bearings or asymmetrical tibial trays, or other surgical techniques. Third, the number of knees studied was small. Although our statistical power was adequate, larger studies with more patients may help to determine how well these findings might generalize to other patients. Fourth, some other physical or psychological patient related factors besides device rotation may have influenced in the clinical result, these factors need to be studied further.

In conclusion, we demonstrated that 2D-CT TKA tibial component rotation determined by the ATA method was the most reliable of those studied. BFA, TE_PTCA and BC_PTCA were reliable methods for TKA femoral and combined rotation. A minimal rotation between the TKA components (BC_PTCA) or a small femoral (BFA) ER or tibial (ATA) IR was useful to predict a successful KSS outcome. There can be variability in TKA rotation measured by the same or different observers; this highlights the difficulty measuring component malrotation and for its clinical use. For that reason, the attribution of symptoms to 2D-CT malrotation requires careful consideration.

## Data Availability

The datasets used and/or analysed during the current study are available from the corresponding author on reasonable request.

## Abbreviations

TKA: Total knee arthroplasty
BMI: Body mass index
ASA: American society of Anesthesiology score
HKA: Hip–knee–ankle axis
2D-CT: Two-dimensional computed tomography
3D-CT: Three-dimensional computed tomography
ER: External rotation
IR: Internal rotation
BFA: Berger femoral angle
BTA: Berger tibial angle
ATA: Anatomic tibial angle
BM_PTCA: Bimalleolar posterior tibial component angle
TE_PTCA: Transepicondylar posterior tibial component angle
BC_PTCA: Bicondylar posterior tibial component angle
TE_BM: Transepicondylar bimalleolar angle
BC_BM: Bicondylar bimalleolar angle
